# Genetically elevated circulating homocysteine concentrations increase the risk of diabetic kidney disease in Chinese diabetic patients

**DOI:** 10.1111/jcmm.14187

**Published:** 2019-02-07

**Authors:** Liang Ma, Qian Liu, Yongwei Jiang, Hailing Zhao, Tingting Zhao, Yongtong Cao, Ping Li, Wenquan Niu

**Affiliations:** ^1^ Clinical Laboratory China‐Japan Friendship Hospital Beijing China; ^2^ Beijing Key Lab Immune‐Mediated Inflammatory Diseases China‐Japan Friendship Hospital, Institute of Clinical Medical Science Beijing China; ^3^ BioBank Center, China‐Japan Friendship Hospital, Institute of Clinical Medical Science Beijing China

**Keywords:** diabetes mellitus, diabetic kidney disease, homocysteine, Mendelian randomization, risk prediction model

## Abstract

Diabetic kidney disease (DKD) is a devastating and frequent complication of diabetes mellitus. Here, we first adopted methylenetetrahytrofolate reductase (*MTHFR*) gene C677T polymorphism as an instrument to infer the possible causal relevance between circulating homocysteine and DKD risk in a Chinese population and next attempted to build a risk prediction model for DKD. This is a hospital‐based case‐control association study. Total 1107 study participants were diagnosed with type 2 diabetes mellitus, including 547 patients with newly diagnosed and histologically confirmed DKD. *MTHFR* gene C677T polymorphism was determined using the TaqMan method. Carriers of 677TT genotype (14.55 μmol/L) had significantly higher homocysteine concentrations than carriers of 677CT genotype (12.88 μmol/L) (*P* < 0.001). Carriers of 677TT genotype had a 1.57‐fold increased risk of DKD (odds ratio: 1.57, 95% CI: 1.21‐2.05, *P* = 0.001) relative to carriers of 677CT genotype after adjusting for confounders. Mendelian randomization analysis revealed that the odds ratio for DKD relative to diabetes mellitus per 5 μmol/L increment of circulating homocysteine concentrations was 3.86 (95% confidence interval: 1.21‐2.05, *P* < 0.001). In the Logistic regression analysis, hypertension, homocysteine and triglyceride were significantly associated with an increased risk of DKD and they constituted a risk prediction model with good test performance and discriminatory capacity. Taken together, our findings provide evidence that elevated circulating homocysteine concentrations were causally associated with an increased risk of DKD in Chinese diabetic patients.

## 1 INTRODUCTION

Diabetes mellitus is common and genetically heterozygous. Globally, 415 million adults aged 20‐79 years had diabetes in 2015 and this number has been projected to reach 642 million by 2040.^1^ The mortality associated with diabetes mellitus is primarily related to both macrovascular and microvascular complications 2. Diabetic kidney disease (DKD) is a devastating and frequent complication of diabetes mellitus, as well as a major cause of end‐stage kidney disease. Divergences in clinical presentation and severity of DKD, along with familial clustering, have provided strong evidence for a genetic component to the disease.^3^, ^4^ Several genome‐wide association studies have been completed with an attempt to identify genetic alterations in predisposition to DKD,^5^, ^6^ the genetic underpinnings and molecular mechanisms, however, remain to be explored. It is widely recognized that intermediate phenotypes usually have greater heritability than the disease itself and so the identification of robust intermediate phenotypes with greater heritability in disease pathogenesis would be desirable.

Elevated concentrations of circulating homocysteine, which is partly under genetic control, were observed to be significantly higher in DKD patients than in simple diabetic patients,^7^, ^8^, ^9^ but this may be influenced by confounding and reverse causation. Mendelian randomization offers a powerful tool to understand causality, particularly for an intermediate phenotype that is controlled by a genetic alternation of relatively strong effect.^10^ Mounting evidence indicates a close relation between circulating homocysteine concentrations and an exonic polymorphism, C677T, in methylenetetrahytrofolate reductase (*MTHFR*) gene, with carriers of 677T allele having elevated homocysteine concentrations.^11^, ^12^, ^13^ However, the implication of long‐term genetically elevated homocysteine concentrations in the development of DKD has not yet been clarified.

To yield more information, we first adopted the C677T polymorphism in *MTHFR* gene as an instrument to infer the possible causal relevance between circulating homocysteine and DKD risk in a Chinese population. We next attempted to build a risk prediction model for DKD by incorporating potential contributing factors.

## 2 MATERIALS AND METHODS

### 2.1 Study participants

This is a hospital‐based case‐control association study conducted at the China‐Japan Friendship Hospital between August 2016 and February 2018. In total, 1107 participants who were diagnosed with type 2 diabetes mellitus were recruited and hospitalized.

Diabetic kidney disease was diagnosed according to the National Kidney Foundation Kidney Disease Outcomes Quality Initiative (NKF‐K/DOQI) guidelines. Patients with type 2 diabetes mellitus who had newly diagnosed and histologically confirmed DKD were classified as the case group (n = 547). The rest 560 patients who had experienced type 2 diabetes mellitus for seven or more years and had no history of DKD and severe kidney diseases formed the control group.

The conduct of this study was approved by the institutional review boards of the China‐Japan Friendship Hospital. All study participants signed informed consent prior to blood sampling for genetic analysis and all of the other procedures associated with this study.

### 2.2 Eligibility criteria

Participants in the case group were included if they had a clinical diagnosis of type 2 diabetes mellitus and 24 hours urinary albumin >500 mg/L or an albumin creatinine ratio (ACR) >30 mg/g and participants were excluded if they had no previous history of kidney diseases or if they had primary or secondary kidney diseases that caused proteinuria, such as IgA nephropathy, membranous nephropathy, lupus nephritis, obstructive renal disease and acute urinary tract infection.

Participants in the control group were included if they had a clinical diagnosis of type 2 diabetes mellitus and ACR <30 mg/g. The exclusion criteria were same as the case group.

### 2.3 Data collection

Each participant was invited to complete a self‐designed structured questionnaire to obtain information on age, sex, bodyweight, body height and smoking habit, hypertension and duration of diabetes mellitus. Body mass index (BMI) was calculated as weight (kg) divided by height squared (m^2^).

Laboratory biomarkers including 24 hours urinary albumin excretion and ACR, high‐density lipoprotein cholesterol (HDLC), low‐density lipoprotein cholesterol (LDLC), total cholesterol (TC), triglyceride, hemoglobin A1c (HbA1c) and homocysteine were assayed. Serum concentrations of fasting triglyceride, TC, HDLC, LDLC and homocysteine were measured using an automated biochemical analyzer (AU5800 Clinical Chemistry System; Beckman Coulter, Brea, CA). HBA1c was measured using the D‐10 Hemoglobin Testing System (Bio‐Rad, Hercules, CA).

### 2.4 Genomic DNA extraction and genotyping

Genomic DNA was extracted from whole blood according to the manufacturer's recommendations and quantified using the NanoDrop 1000 spectrophotometer (ThermoScientific). DNA samples were frozen at −20°C until the time of analysis.

*MTHFR* gene C677T polymorphism was determined using the TaqMan SNP Genotyping Assay (Applied Biosystems) using the primer sequences: F: 5′‐GGC TGA CCT GAA GCA CTT GAA‐3′ and R: 5′‐AGA AAA GCT GCG TGA TGA TGA A‐3′. The probe sequences were: FAM‐5′‐TCT GCG GGA GTC G‐3′‐MGB; VIC‐5′‐CTG CGG GAG CCG A‐3′‐MGB. The primers and probes were designed by Applied Biosystems.

Fifty nanograms of DNA was amplified in a 25 μL reaction mixture containing 12.5 μL of Premix Ex Taq (Takara, Shiga, Japan), 5 pmol of each primer (Applied Biosystems) and 3 pmol of each probe (Applied Biosystems) for the amplification of *MTHFR* genomic sequence. Pre‐heating of the mixture at 95°C for 10 minutes followed by 40 cycles of denaturation at 95°C for 15 seconds and then by annealing and elongation at 65°C for 60 seconds.

To verify the genotypes, 50 polymerase chain reaction (PCR) products were randomly selected for DNA sequencing using the ABI 3500 Genetic Analyzer (Applied Biosystems) and the results were 100% concordant.

### 2.5 Statistical analysis

Continuous variables were expressed as mean (SD) and categorical variables as number (percentage). Two group comparisons were performed using the *t* test or Wilcoxon rank‐sum test or Chi‐squared test where appropriate. Pearson correlation analysis was conducted to examine the relevance between homocysteine and lipid biomarkers. Forward Logistic regression analysis was used to select potential contributing factors at a significance level of 5%.

The −2 Log likelihood ratio test was used to compare the fit of two models. The goodness of fit of the model was justified using the Hosmer‐Lemeshow test. The receiver operating characteristic (ROC) curves were plotted for models with and without significant factors.

The Sobel‐Goodman mediation test was performed to test whether a mediator carried the influence of homocysteine on DKD risk. The net benefits of adding significant factors to basic model were seen by using decision curve analysis.^14^ Finally, a nomogram was plotted for significant factors using regression modelling strategies (rms) program in the r software version 3.5.0.

A two‐sided *P* less than 0.05 was considered the threshold of statistical significance. Unless otherwise stated, statistical analysis was completed using the stata/se software version 14.0 (StataCorp., College Station, TX).

## 3 RESULTS

### 3.1 Baseline characteristics

Table 1 shows the baseline characteristics of the study participants. More males were found in the case group than in the control group (67.09% vs 61.03, *P* = 0.035), as well as for hypertension percentage (77.27% vs 52.85%, *P* < 0.001). Mean levels of BMI, triglyceride and homocysteine were significantly higher in the case group than in the control group. The genotype distributions of *MTHFR* gene C677T polymorphism differed significantly between the two groups (*P* < 0.001), with the 677TT genotype overrepresented in the case group.

**Table 1:** Baseline characteristics of study participants

Characteristics	Case group (n = 547)	Control group (n = 560)	*P*
Age (y)	62.51 (11.45)	61.52 (10.17)	0.137
Sex (males)	369 (67.09%)	343 (61.03%)	0.035
BMI (kg/m^2^)	26.30 (3.65)	25.67 (3.36)	<0.001
Smoking	205 (37.27%)	185 (32.92%)	0.128
DM duration (y)	15.11 (8.11)	14.90 (6.02)	0.582
Hypertension	425 (77.27%)	297 (52.85%)	<0.001
TG (mmol/L)	2.17 (1.74)	1.84 (1.44)	<0.001
TC (mmol/L)	4.38 (1.46)	4.24 (1.08)	0.394
HDLC (mmol/L)	1.01 (0.33)	1.04 (0.30)	0.041
LDLC (mmol/L)	2.52 (0.95)	2.51 (0.81)	0.663
HbA1c (%)	8.14 (1.81)	8.12 (1.74)	0.922
Homocysteine (μmol/L)	14.61 (6.17)	12.36 (4.77)	<0.001
*MTHFR* gene C677T genotypes			<0.001
CC	95 (17.37%)	71 (12.68%)	
CT	255 (46.62%)	328 (58.57%)	
TT	197 (36.01%)	161 (28.75%)	

Data are expressed as mean (SD) for continuous variable and number (percentage) for categorical variables. *P* value was calculated using the *t* test or Wilcoxon rank‐sum test or Chi‐squared test where appropriate.

BMI, body mass index; DM, diabetes mellitus; HbA1c, hemoglobin A1c; HDLC, high‐density lipoprotein cholesterol; LDLC, low‐density lipoprotein cholesterol; MTHFR, methylenetetrahytrofolate reductase; TC, total cholesterol; TG, triglyceride.

### 3.2 Genotype‐phenotype association

Carriers of the 677TT genotype (14.55 [5.89] μmol/L) had significantly higher homocysteine concentrations in circulation than carriers of the 677CT genotype (12.88 [5.19] μmol/L) (*P* < 0.001), whereas there was no significance relative to the 677CC genotype (13.30 [6.18] μmol/L) (*P* = 0.058).

### 3.3 Genotype‐disease association

Carriers of the 677TT genotype had 1.57‐fold increased risk of DKD (odds ratio: 1.57, 95% CI: 1.21‐2.05, *P* = 0.001) relative to carriers of 677CT genotype after adjusting for confounding factors (age, sex, BMI, smoking, hypertension, duration of diabetes mellitus, triglyceride, TC and HDLC), whereas no significance was found for the comparison of the 677TT genotype vs 677CC genotype.

### 3.4 Correlation between homocysteine and four lipids

**Figure 1:**
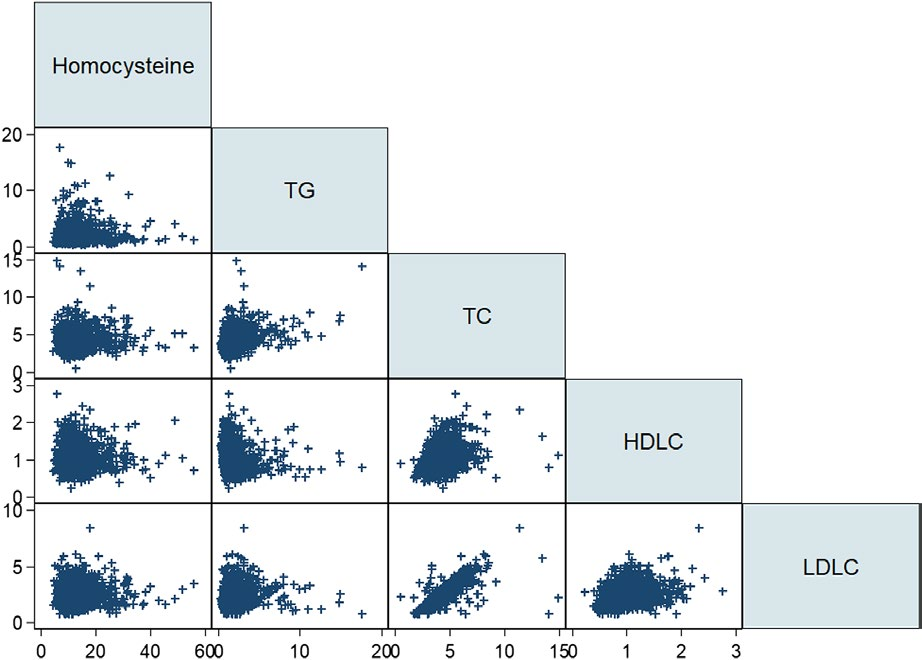
Correlation of homocysteine with four blood lipids

Figure 1 presents the correlation plot of homocysteine and four blood lipids. The correlation coefficient of circulating homocysteine with lipids ranged from 0.006 to 0.027, with no detectable significance. In addition, the Sobel‐Goodman mediation test failed to reveal any significant contribution of BMI and four blood lipids to the relevance between circulating homocysteine concentrations and DKD risk (Table S1).

### 3.5 Mendelian randomization estimate

Based on the risk estimates of genotype‐phenotype and genotype‐disease associations and under the rationales of Mendelian randomization approach, the odds ratio for DKD relative to diabetes mellitus per 5 μmol/L increment of circulating homocysteine concentrations was 3.86 (95% confidence interval: 1.21‐2.05, *P* < 0.001) implying a potential causal role of homocysteine in the pathogenesis of DKD.

### 3.6 Selection of significant factors

Significant factors in association with DKD risk were selected based on the Forward Logistic regression analysis (Table 2). Three factors, hypertension, homocysteine (+5 μmol/L) and triglyceride, were significantly associated with an increased risk of having DKD (odds ratio: 2.87, 1.39 and 1.12, 95% confidence interval: 2.18‐3.77, 1.21‐1.58 and 1.03‐1.22, *P* < 0.001, <0.001 and =0.009 respectively).

**Table 2:** Selected variables of statistical significance in association with diabetic kidney disease in the Forward Logistic regression analysis

Significant variables	Odds ratio	95% confidence interval	*P*
Hypertension	2.87	2.18‐3.77	<0.001
Homocysteine (+5 increment)	1.39	1.21‐1.58	<0.001
Triglyceride	1.12	1.03‐1.22	0.009

### 3.7 Prediction performance assessment

Prediction models with (full model) and without (basic model) three significant factors differed significantly and both models showed goodness of fit (Table 3).

**Table 3:** Performance tests for adding three significant variables to basic model in prediction of diabetic kidney disease

Tests	Basic model^a^	Full model^b^
−2 Log likelihood ratio test		
Chi‐squared	94.86	
*P*	<0.001	
Hosmer‐Lemeshow test		
*P*	0.323	0.298
ROC comparison		
Area under the ROC curve	0.705	0.798
95% CI	0.671‐0.739	0.767‐0.829
*P* for area difference	<0.001	

95% CI, 95% confidence interval; ROC, receiver operating characteristic.

a Basic model included age, sex, body mass index, smoking, duration of diabetes mellitus, total cholesterol, high‐density lipoprotein cholesterol, low‐density lipoprotein cholesterol, hemoglobin A1c and *MTHFR* gene C677T polymorphism.

b Full model: basic model plus three significant factors (hypertension, homocysteine and triglyceride).

### 3.8 Decision curve analysis and nomogram

**Figure 2:**
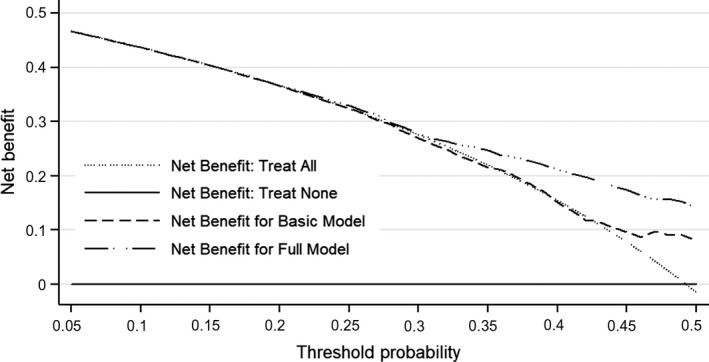
Decision curve analysis on the net benefits of adding three significant factors to the basic model. Full model included basic model and three significant variables (hypertension, homocysteine and triglyceride)

The benefits gained by adding three significant factors to the basic model were higher than the benefits of the basic model (Figure 2).

**Figure 3:**
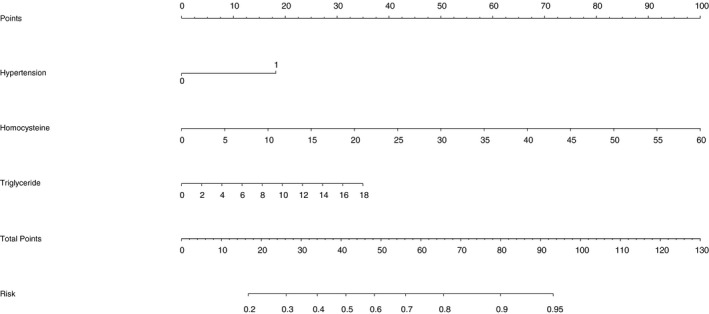
Nomogram calculator of three significant factors in prediction for diabetic kidney disease

Based on three significant factors, a nomogram is plotted in Figure 3 to predict the risk of DKD relative to diabetes mellitus. The C‐index of this nomogram to assess prediction accuracy was 0.71 (*P* < 0.01) indicating good prediction performance.

## 4 DISCUSSION

Our findings indicated that *MTHFR* gene C677T polymorphisms were significantly associated with circulating homocysteine changes and DKD risk in Chinese diabetic patients. Under the principles of Mendelian randomization, we found that elevated circulating homocysteine concentrations were causally associated with an increased risk of DKD when using the C677T polymorphism as an instrumental variable. Additionally, circulating homocysteine, annexed with hypertension and triglyceride constituted a powerful prediction model for DKD risk and this model had good test performance and discriminatory capability. To our knowledge, this is the first Mendelian randomization study that has provided support for a causal role of circulating homocysteine in the pathogenesis of DKD.

Homocysteine is a sulphur‐containing non‐proteinogenic amino acid biosynthesized from methionine and abnormal elevation of circulating homocysteine concentrations has been implicated in the development of many clinical end‐points or pathological conditions, such as cardiovascular disease,^15^ stroke^16^ and microalbuminuria.^17^ Hyperhomocysteinaemia is an independent risk factor for glomeruloslerosis and renal insufficiency and the association of circulating homocysteine with DKD was widely evaluated in the medical literature, yet the results are not often reproducible.^7^, ^8^, ^9^, ^18^, ^19^, ^20^, ^21^, ^22^ Actually, current evidence linking homocysteine to DKD is mainly based on observational data, in which the degree of possible confounding and reverse causation may cloud the true relationship.^23^, ^24^ In this context, Mendelian randomization has proven to be a valuable method to overcome confounding and reverse causality^25^, ^26^ and this method enables estimation of causal relationship in observational studies using genetic alterations as instruments.^27^ Following the principles of Mendelian randomization, we found that per 5 μmol/L increment in circulating homocysteine concentrations increased approximately four‐fold the odds of having DKD. This finding was relatively convincing, as the instrumental polymorphism (C677T) selected in this study was simultaneously and significantly associated with circulating homocysteine changes and DKD risk by many studies^11^, ^28^, ^29^, ^30^, ^31^ and further the association between homocysteine and DKD risk was not mediated by obesity and blood lipids. For practical reasons, there is potential clinical utility in the consideration of homocysteine concentrations among diabetic patients. In support of our findings, peroxisome proliferator‐activated receptor gamma (PPAR‐γ) agonist ciglitazone can protect DKD in part by activating PPAR‐γ and clearing glomerular tissue homocysteine.^32^ Pending prospective reproducible investigations circulating homocysteine concentrations can help identify diabetic patients with a high risk of DKD who could benefit from closer monitoring.

The underlying mechanisms for the contribution of increased circulating homocysteine concentrations to DKD risk are not fully understood, although several explanations have been proposed. First, homocysteine can result in vascular damage, such as endothelial dysfunction, cell proliferation, increased oxidative stress and prothrombotic state^33^ and high total homocysteine concentrations may potentially induce renal injury via direct action on kidney cells, rather than cause impaired renal function, which at least in part supports the fact that impaired renal function reduces renal clearance of homocysteine. Second, extrarenal metabolism defects in homocysteine or intrarenal defects can result in increased circulating homocysteine concentrations in patients with DKD. As reported by Van Guldener and colleagues, homocysteine remethylation, the main metabolized pathway in homocysteine degradation, was diminished in patients with ESRD related to healthy controls.^34^ Third, there is evidence that *MTHFR* gene C677T mutation and low dietary intake of folate were associated with increased circulating homocysteine concentrations.^35^

Although our findings support that long‐term genetically elevated homocysteine can result in an increased causal risk for DKD, we further attempted to build a risk prediction model, as the process of DKD development is complex and involved many risk factors. Toward this goal, we employed a comprehensive panel of analytical methods and demonstrated that homocysteine, together with hypertension and triglyceride can better predict the future risk of DKD among diabetic patients. Importantly, this model had good test performance and discriminatory capacity. According to the weight of each element in this model, homocysteine played a leading role in decision making about the magnitude of DKD risk highlighting the importance of homocysteine in the pathogenesis of DKD. Nevertheless, we agree that it is of clinical importance to replicate this risk prediction model in other independent groups and refine risk‐assessment algorithms with more desired accuracy.

Finally, it is important to consider the potential limitations of this study. First, we recruited study participants at a mono‐site, which may limit the generalizability of our findings, although it facilitates consistency of diagnosis and evaluation. Second, only one polymorphism in *MTHFR* gene was selected as an instrumental variable and it is of added interest to interrogate the suitability of other functional polymorphisms, such as A1298C.^11^ Third, given the small number of study participants, we were unable to perform further subgroup analyses such as by sex and smoking habit. Fourth, we only tested the mediation effect of obesity and four blood lipids and it is unclear whether other important contributing factors might mediate the association of circulating homocysteine with DKD risk. Moreover, it would be more interesting to detecting homocysteine concentrations in urinary samples, which are not available for us. Fifth, our findings were exclusively derived from a Chinese population and external replication would be necessary.

Despite these limitations, our findings provide evidence that elevated circulating homocysteine concentrations were causally associated with an increased risk of DKD in Chinese diabetic patients. Moreover, we have built a powerful risk prediction model, including homocysteine, hypertension and triglyceride, which can allow early detection and targeted treatment in the control of DKD. For practical reasons, we hope this study will not remain just another end‐point of research instead of a start to establish fundamental data to further explore the molecular mechanisms of circulating homocysteine and DKD.

## CONFLICT OF INTEREST

The authors declare that they have no competing interests.

## AUTHOR CONTRIBUTIONS

Conceptualization and Draft writing: Liang Ma, Yongtong Cao, Ping Li, Wenquan Niu. Data curation: Liang Ma, Qian Liu, Yongwei Jiang, Hailing Zhao, Tingting Zhao, Wenquan Niu. Funding acquisition: Liang Ma. Investigation: Liang Ma, Qian Liu, Yongwei Jiang. Data detection: Liang Ma, Hailing Zhao, Tingting Zhao. Statistical analysis: Wenquan Niu.
