# Supplementary material for: Genetically elevated circulating homocysteine concentrations increase the risk of diabetic kidney disease in Chinese diabetic patients
Source: J Cell Mol Med. 2019 Feb 7;23(4):2794–800. doi: 10.1111/jcmm.14187 (PMC6433716; doi:10.1111/jcmm.14187)
Supplement: Supplementary file 1 [file JCMM-23-2794-s001.pdf]

Supplementary Table 1. Sobel-Goodman mediation tests for the association of homocysteine with diabetic kidney disease.

**Mediators**	**P for Sobel**	**P for Goodman-1**	**P for Goodman-2**
BMI	0.432	0.450	0.411
TG	0.403	0.422	0.382
TC	0.436	0.488	0.365
HDLC	0.836	0.869	0.749
LDLC	0.969	0.979	NA

Abbreviations: BMI, body mass index; HbA1c, hemoglobin A1c; TG, triglyceride; TC, total cholesterol; HDLC, high-density lipoprotein cholesterol; LDLC, low-density lipoprotein cholesterol; NA, not available.
